# Technical note: Construction of a CO_**2**_ supply system for depopulation of pigs in a container

**DOI:** 10.1093/tas/txaf034

**Published:** 2025-03-11

**Authors:** Marianne Kaiser, Jens Kristian Kristensen, Peter T Thomsen

**Affiliations:** Department of Animal and Veterinary Sciences, Aarhus University, Tjele 8830, Denmark; Department of Electrical and Computer Engineering, Aarhus University, Aarhus N 8200, Denmark; Department of Animal and Veterinary Sciences, Aarhus University, Tjele 8830, Denmark

**Keywords:** carbon dioxide, disease control, emergency, gas, killing, livestock

## Abstract

Situations may arise where authorities need to depopulate large quantities of pigs in a short time. This must be done in an animal welfare-responsible manner. This paper describes in detail a technical mobile container system for CO_2_ depopulation of pigs. The system consists of simple and easily accessible materials and can be replicated and scaled for multiple container systems for CO_2_ depopulation. The container system was tested at 4 depopulation events where the pigs’ behavior was filmed (1 event) and the duration of the various procedures was recorded. The results showed that the system’s capacity met AVMA’s recommendations for a CO_2_ supply rate of 10% to 30% of the chamber volume per min. On average, the containers were supplied with CO_2_ for 7 min, and it was possible to maintain a CO_2_ concentration of 80% for at least 10 min after stopping the CO_2_ admission. By maintaining the tarpaulin on the container during transport, this “extending effect period” can be utilized for additional CO_2_ exposure and acts as an extra safeguard for successful depopulation. Target CO_2_ concentration of 80% occurred after a mean of 7 min. Pig escape attempts were first observed 2 min:26 s after the start of CO_2_ exposure. Presumably due to the stocking density (a mean of 0.52 m^2^ per pig), no loss of posture (indicating loss of consciousness) could be observed. On the other hand, the last escape attempts were observed after 3 min:04 s, and the last atactic movements after 3 min:13 s. It is therefore reasonable to assume that all pigs have lost consciousness around that time. No pigs survived the procedure, and the described CO_2_ depopulation system therefore lived up to expectations. We recommend that users are given the opportunity to practice thoroughly before an authentic emergency.

## Teaser

Technical details of a mobile CO_2_ depopulation system for pigs are described. The system consists of easily accessible components and can be produced quickly in multiple numbers in emergency situations.

In case of outbreaks of critically infectious diseases, the Danish Veterinary and Food Administration must be able to depopulate large numbers of pigs at short notice at all kinds of farms. Such a method must be acceptable in terms of animal welfare. For efficient depopulation of pigs, a constant CO_2_ supply of 80% to 90% for a minimum of 5 min is necessary (AVMA, 2013). In addition, the AVMA recommendations (2013) state a displacement rate of 20% of the chamber volume per min. corresponding to a CO_2_ concentration of 85.5% in 10 min. Also, monitoring the pigs during depopulation is essential to be able to implement immediate corrective measures if necessary (Gavinelli et al., 2014). This is the background for this detailed description of a CO_2_ depopulation system for pigs commissioned by the Danish authorities in 2022. The system seeks to enable overall control points such as live animal handling, depopulation efficiency and humaneness, as well as death confirmation as previously suggested by Berg (2012). This can support the authorities in realistic and reliable scenario planning.

The system consists of a mobile container system that can be quickly established in large numbers with cheap and readily available equipment. The container system is compatible with requirements described by Stikeleather et al. (2013) , whose simulations demonstrated how CO_2_ was distributed uniformly 25 cm above the floor in an undivided container with finishers. Also, the system has similarities with the system described by Meyer and Morrow (2005), who released CO_2_ directly from a commercial liquid CO_2_ delivery truck into a container, but it also differs from this in significant respects. To our knowledge, a CO_2_ depopulation system like the present has never been presented before.

At the time of writing, new projects investigating the effect of gases added to water-based foam (e.g., Lindahl et al., 2020; Korenyi-Both et al., 2022; Campler et al., 2023) as an alternative depopulation method have been described. The technique seems promising (Arruda et al., 2020) but was not chosen here, as it has not yet been approved for pigs, and the authorities needed a reliable depopulation system which can be used at the present time. Thus, the presented method is still relevant. The use of other gases for depopulation will not be discussed in this paper. Finally, the behavior and physiology of the pigs during depopulation are only briefly described, as this topic will be elaborated on in a follow-up publication.

## MATERIALS AND METHODS

### Animals

The pigs used in this project were offspring of sows that had participated in a study conducted at the Department of Animal and Veterinary Sciences, Aarhus University, Tjele, Denmark. The study complied with current regulations on animal experiments, and the protocol was approved by the Danish Animal Experiments Inspectorate, ID number: 2018-15-0201-01484.

In total, 101 finishers (25, 25, 27 and 24 pigs respectively) in the weight range 73 to 136 kg (mean weight 105 kg) were depopulated in containers on four occasions. The finishers were DanBred crosses (D-LY) of both sexes (sows and boars) housed in the university finisher barn.

### Container Design

**Figure 1.**
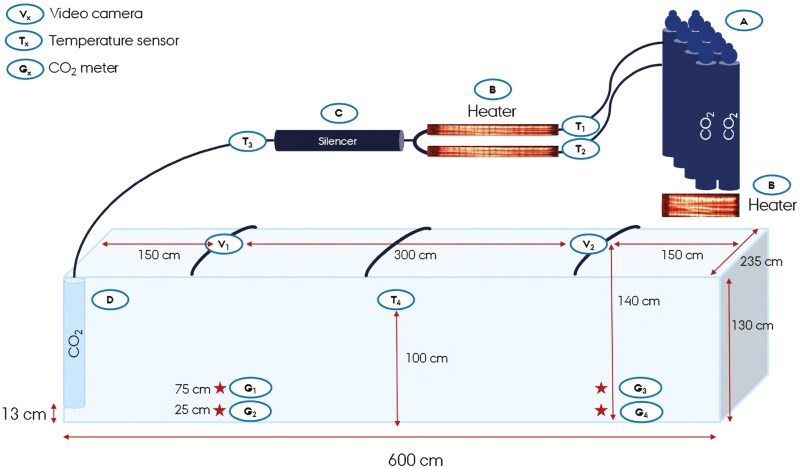
Sketch of a container for depopulation of pigs. Sketch of a container for depopulation of larger groups of pigs. CO_2_ is supplied from gas cylinders (A) after heating with heaters (B) located in two different places in the installation. After heating, the gas is led through a silencer (C) after which it is blown through a 110 mm ⌀ pipe (D) that opens 13 cm above the floor height of the container. The container was equipped with two video cameras (V1-2), four temperature sensors (T1-4) and four CO_2_ meters (G1-4) for monitoring the process and the pigs’ behavior (Drawing: Kaiser, AU).

A low mobile container (600 × 130 × 235 cm inside; Micodan, Støvring, Denmark) equipped with a dark blue tarpaulin (660 g per m^2^; Micodan, Støvring, Denmark) was used for depopulation. A sketch of the container’s design and dimensions is shown in Figure 1. The container was provided with a two-part door in one end. Under the tarpaulin, three curved iron bars were placed across to divert rainwater and allow attachment of two video cameras (V1-2; DS-2CDD2045FWD-I with 2.8 mm lens, Hikvision, Hangzhou, China). The distance from the video cameras to the bottom of the container was 140 cm, corresponding to the highest point under the tarpaulin. The distance from the cameras to the end of the container was 150 cm (Figure 1).

To fill the container, two CO_2_ batteries (Carbon Dioxide Battery 240 kg; Air Liquide Danmark A/S, Taastrup, Denmark) were used consisting of 16 gas cylinders in total. To prevent freezing, the cylinders were heated by 8 radiant panels (Terrassevarmere 1912; Harald Nyborg, Odense SV, Denmark) placed below the CO_2_ batteries. Each radiant panel had an output of 1,500 W, corresponding to 6 kW per battery with 8 gas cylinders. The cylinders reached a maximum temperature of 55 °C under the bottom, when the sidewall was 25 °C, which was considered safe and approved by the CO_2_ supply company (Air Liquide Danmark A/S, Taastrup, Denmark). From the gas cylinders, CO_2_ was led through two hoses (⌀ 2.54 cm) to two tubes (⌀ 3.81 cm), each of which contained a 2,000 W heater (1½“ El cartridge; Klingenberg Electronics, Odense, Denmark). To limit the pigs’ stress burden caused by the CO_2_ blow-in, a silencer was implemented between the gas heaters and the gas hose leading to the container. The silencer consisted of a 1 m long plastic pipe (Nicoll HTP drainpipe with sleeve ⌀ 110, 100 mm; Nicoll Nordic, Copenhagen K, Denmark) lined with a 48 × 30 mm Rockwool pipe bowl (Rockwool Danmark A/S, Hedehusene, Denmark). After passing the silencer, CO_2_ was blown through a flexible hose (Suction/pressure hose Luisana SE ⌀ 50, Maykers, Skjern, Denmark) and thereafter through a steel pipe (⌀ 110 mm; Blücher, Vildbjerg, Denmark). The steel pipe opened in the left corner of the container’s front, ending 13 cm above the floor (Figure 1).

The gas temperature was measured before it passed the last heaters and after the silencer (T1-3), as well as in the center of the container (T4), with temperature sensors (Type T, RS PRO, RS no. 762-1146, RS Components). Four CO_2_ meters (G1-4; Geotech G110, Q-ED Environmental Systems, Coventry, UK) were placed 150 cm from the front and rear end, respectively, and 25 cm and 75 cm, respectively, above the floor (Figure 1). A detailed description of the container’s design is presented as Supplemental Material. During the depopulation of pigs, the ambient climate was recorded approx. 1 km from the container (Foulum Klimastation, Blichers Allé 20, Tjele, Denmark).

### Study Design

Prior to the depopulation of live pigs, several test measurements were carried out in empty containers until the supply rate and CO_2_ temperature met the AVMA’s recommendations corresponding to a supply rate of 10% to 30% CO_2_ per minute of a given chamber volume (Rice et al., 2014). To test the risk of skin damage caused by frostbite, a pilot study was carried out with 2 dead pigs and later 2 anesthetized pigs placed as close as possible to the CO_2_ blow-in pipe (about 5 cm). No frost damage could be observed on the pigs’ skin. In addition, the temperature in the area was never measured below 0 °C.

The finishers were depopulated on 4 occasions in February (Container 1) and March 2023 (Container 2 to 4). The day before depopulation, the finishers were weighed in a single animal scale (Schauer SDU TS4D; Schauer Agrotronic GmbH, Prambachkirchen, Austria) for calculation of the stocking density (mean of 0.52 m^2^ per pig), which followed the Danish requirements for pigs during transport over 8 h (Fødevareministeriet, 2020). This stocking density was chosen since this density table is well defined in the Danish legislation and requires that all pigs as a minimum be able to lie down and stand up in their natural position plus an area allowance of 20%. On the day of depopulation, the container floor was provided with sawdust mixed with pig feed and the finishers were loaded directly from the barn’s corridor. At the second depopulation event, the three batches of finishers were depopulated immediately after each other using two containers to test the flow and efficiency in a more hectic environment. The time of important procedures (e.g., closing of container and start time of CO_2_ injection) was recorded continuously at all events.

Supply of CO_2_ began immediately after the last pig had entered the container and ended when the last of the four CO_2_ meters showed a CO_2_ concentration of 80%. After the end of the CO_2_ supply, a 10 min break was scheduled to investigate whether a closed tarpaulin could maintain a CO_2_ concentration above 80% for an extended period, which could function as an extra safety measure that could be utilized during transport in an authentic depopulation scenario. After the 10 min break, the tarpaulin was removed, and the container moved approx. 500 m for emptying and verification of all pigs’ death. A sample of 38 randomly selected pigs was checked for the absence of heartbeat, respiration, palpebral reflex and corneal reflex. For practical reasons, the remaining pigs were only verified dead by auscultation of the heart.

The pigs’ behaviors in Container 1 (the weather conditions caused the camera lenses to steam up in container 2 to 4) were analyzed continuously (Lehner, 1992) according to a modified version of Sadler et al. (2014) (See Table 1). This was done by a trained observer using the software program BORIS v. 8.20.3. (Friard et al., 2016). Due to high stocking density, it was only possible to record the first and last time a given behavior appeared, and only standing, sitting and lying positions, atactic movement, muscular excitation, escape attempts and last movement could be observed.

**Table 1.** An ethogram for the behavior of pigs depopulated with CO_2_ in a container. All variables were analyzed continuously but due to high stocking density, only the first and the last time a given variable was observed was registered. The ethogram is modified after Sadler et al. (12).

**Behavior** **States**		
Standing up	The body weight of the pig is on the feet with the legs extended or legs in movement in an upright position.	min:s from CO_2-start_
Sitting	The pig sits with the hindquarters/rear part of the body resting against the ground and the forelegs stretched	min:s from CO_2-start_
Lies	The pig does not bear weight on its limbs and the condition is maintained for the rest of the observation period. Maintenance of lying position.	min:s from CO_2-start_
**Incidents/Events**		
Escape attempt	The pig raises its front legs (one or both) on the side of the container wall or pushes quickly and forcefully with its head or snout on the sides of the container; strong coordinated movement towards the outside of the container; events within a period of 10 s. is registered as a single escape attempt.	Number of times and min:s from CO_2-start_
Atactic movement	The pig moves in an apparently uncoordinated manner; lack of muscle coordination when the pig performs voluntary movements	min:s from CO_2-start_
Muscular excitation	Repetitive muscle movements of the whole body, including upward movements of the head; apparently uncoordinated (categorization of number of excitations as well as posture is not possible due to rapid and frequent movements); severe excitation appears as major clonic convulsive attacks.	min:s from CO_2-start_
Last movement	No movement of any kind is observed from the pig after this movement	min:s from CO_2-start_

## RESULTS AND DISCUSSION

### CO_2_ Supply

We experienced no technical problems during any of the four depopulation events and the capacity met AVMA’s recommended CO_2_ supply rate of 10% to 30% of the chamber volume per min (Rice et al., 2014). With the chosen exposure times for CO_2_ concentrations of 80% for all CO_2_ meters, no pigs were alive at the end of the procedure, and no skin damage caused by frostbite was observed. The CO_2_ supply was stable and consistent under all conditions.

 Technical data for the four depopulation events are listed in Table 2. The mean loading time was 5 min:45 s, it took 1 min:15 s to close the containers and thereafter 1 min:15 s until the CO_2_ supply started. The containers were supplied with CO_2_ for a mean of 7 min. It was possible to maintain a CO_2_ concentration of at least 80% for all CO_2_ meters during the CO_2_ exposure period, which included an “extended effect period” of 10 min with closed tarpaulin and no CO_2_ supply. On average, it took 23 min from CO_2_ supply start until the tarpaulin was removed (Table 2).

**Table 2.** Technical data for loading, CO_2_ supply and verification of death of 101 finishers exposed to CO_2_ in 4 containers.

Container number	1	2	3	4	Avg.
Number of pigs	25	25	27	24	25.3
Total pig weight pr container (kg)	2,711	2,619	2,670	2,639	2,659.8
Average weight of pigs (kg)	108.4	104.8	98.9	110	105.5
Stocking density (Number of m^2^ per pig)	0.531	0.517	0.496	0.534	0.520
From the 1st pig was outside the barn to the end of loading (min)	7	5	6	5	5:45
Closing the container and attaching the tarpaulin (min)	2	1	1	1	1:15
From closure of the tarpaulin to start of CO_2_ supply (min)	1	1	1	2	1:15
CO_2_ meter 1: Time from CO_2_ supply started to CO_2_ concentration of 40% (min:sec)	2:00	2:30	1:15	1:15	1:45
CO_2_ meter 2: Time from CO_2_ supply started to CO_2_ concentration of 40% (min:sec)	2:30	2:00	1:30	1:45	1:45
CO_2_ meter 3: Time from CO_2_ supply started to CO_2_ concentration of 40% (min:sec)	3:00	3:00	2:15	2:30	2:41
CO_2_ meter 4: Time from CO_2_ supply started to CO_2_ concentration of 40% (min:sec)	3:00	3:00	2:30	2:30	2:45
CO_2_ meter 1: Time from CO_2_ supply started to CO_2_ concentration of 80% (min:sec)	6:45	6:00	4:30	4:30	5:30
CO_2_ meter 2: Time from CO_2_ supply started to CO_2_ concentration of 80% (min:sec)	6:45	6:00	4:30	5:00	5:30
CO_2_ meter 3: Time from CO_2_ supply started to CO_2_ concentration of 80% (min:sec)	7:30	7:45	6:45	6:45	7:15
CO_2_ meter 4: Time from CO_2_ supply started to CO_2_ concentration of 80% (min:sec)	8:30	7:00	5:00	5:30	6:30
Average temperature in the center of the container (T4) during the CO_2_ supply (°C)	11.3	21.3	22.2	21.0	19.0
Lowest observed temperature (°C)	8,8	20,1	20,2	19,1	17,1
Highest observed temperature (°C)	15,6	22,8	24,7	22,8	21,5
Total time for CO_2_ supply (min)	9	7	6	6	7
From the start of CO_2_ supply until the tarpaulin was removed (min)	36	20	18	18	23
From the 1st pig was outside the barn until the tarpaulin could be removed—excl. a transport of approx. 500 m (min:sec)	46	27	26	26	31:15
From the removal of the tarpaulin until 10 pigs were declared dead—incl. a transport of approx. 500 m (min:sec)	15	17	14	15	15:1

Previously described comparable depopulation systems (Meyer and Morrow, 2005; Rice et al., 2014; Kinsey et al., 2016; Pepin et al., 2022) are designed slightly differently, and the exact location of CO_2_ meters is not stated, which makes the efficacy of the different systems difficult to compare. In Rice et al. (2014), CO_2_ was transferred from high-pressure batteries to a low-pressure intermediate tank before finally being blown into the depopulation chamber. This procedure ensures a fast feed rate without risk of cooling down the CO_2_. Rice et al. (2014) achieved a CO_2_ concentration of 40% after approx. 1 min:40 s, which roughly corresponds to the values we measured with CO_2_ meter 1 (1 min:45 s; Table 2). Kinsey et al. (2016) and Pepin et al. (2022) also used low-pressure intermediate tanks. Kinsey et al. (2016) achieved a CO_2_ concentration of approx. 80% after 5.2 min. Pepin et al. (2022) validated a standard dump trailer converted into a mobile unit for depopulation of finishers and sows. The trailer was built with a ceiling height of 1 m and CO_2_ was supplied from an intermediate tank through four hoses inserted into the lower side wall. This trailer was filled at a flow of 17% to 20%/min. We chose not to use low-pressure intermediate tanks as such equipment is expensive to manufacture and keep in stock and takes up a lot of space at the depopulation site. Instead, we heated the CO_2_ cylinders and the gas pipes continuously to avoid them cooling down. Our system is characterized by components that can be quickly acquired for production of multiple units.

Meyer and Morrow (2005) described two depopulation methods for pigs using 1) three unregulated gas cylinders and 2) a gas bulk tanker for CO_2_ supply of a dump body trailer. The technical details were sparsely described whereby the method is difficult to compare to ours. However, a Danish supplier of CO_2_ (Air Liquide, Taastrup, Denmark) advised against a system using CO_2_ bulk tanks due to a potential high risk of unstable CO_2_ supply caused by cooling during evaporation of CO_2_.

A human communication error unnecessarily delayed the process of our 2^nd^ depopulation which caused the period of CO_2_ supply to be slightly longer, and as shown in Table 2, the CO_2_ supply time was less for the last two containers compared to containers 1 and 2. This indicates that operators need to be trained in using the CO_2_ depopulation system. For reference, it is difficult to achieve a completely uniform supply rate between the different CO_2_ fillings since the CO_2_ supply rate depends on how full the CO_2_ cylinders are. A full CO_2_ cylinder has higher pressure and therefore the gas will be colder during the supply than partially empty cylinders. A full cylinder will also require more heat input to the gas than an almost empty cylinder, delaying the process. Conversely, the CO_2_ supply will become slightly faster as the cylinders are depleted. When the CO_2_ cylinders are almost empty, the gas pressure drops to such an extent that the supply slows down again. We therefore recommend that authorities and other users have had the opportunity to practice thoroughly before actual depopulation events begin, as also suggested by Gavinelli et al. (2014).

### Behavior

Behavioral assessment was only done for container 1, as the weather conditions during depopulation in container 2 to 4 caused the camera lenses to steam up. It was also not possible to assess individual behavior due to high stocking density, which made the video analysis very difficult. Instead, the pigs’ behavior was evaluated for the group as a unified whole and the results of the video observations can be seen in Table 3.

**Table 3.** First and last time a given behavior appeared in a group of pigs (n = 25) which were depopulated with CO_2_ in a container. The assessment of the pigs’ behavior began from the start of CO_2_ supply. The observations are collected from two cameras placed above the pigs.

Behavior	First observation	Last observation
	(min:sec)	(min:sec)
Standing	0:00	3:12
Sitting	2:44	3:21
Lies^*^	-	3:22
Atactic movement	2:33	3:13
Muscular excitation	2:39	7:34
Escape attempt	2:26	3:04

^*^One pig was lying down from the start of the CO_2_ supply.

An early reaction to CO_2_ exposure is escape attempts, indicating consciousness among the pigs. For comparison with our results, showing escape attempts 2 min:26 s after CO_2_ exposure, Kinsey et al. (2016) observed escape attempts already within the first 33 s. The differences between systems could be explained by a delayed CO_2_ exposure in our design compared to systems using low pressure intermedia tanks.

In the group of pigs, we observed periodic atactic movements in the period approximately 2 to 3 min and muscular excitations in the period approximately 2½ to 7½ min after CO_2_ supply started. For atactic movements, the pigs move in an apparently uncoordinated manner with a lack of muscle coordination, but still with voluntary movement, which indicates the pigs are still conscious. This is consistent with Sadler et al. (2014) who recorded ataxia in 99% of pigs before loss of posture. Loss of posture is considered a good indicator of loss of consciousness (Llonch et al., 2013; Verhoeven et al., 2016) and according to Raj and Gregory (1995), this behavior occurs at CO_2_ concentrations of around 40%. Lying and loss of posture are, however, not comparable variables, since lying only indicates that pigs no longer stand whereas loss of posture refers to a specific behavior where the pig is unable to stand. Presumably due to high pig stocking density, we were not able to recognize loss of posture in the pig group and therefore not able to correlate this behavior with the time of 40% CO_2_ concentration. Since the current project demonstrated escape attempts and atactic movements for the last time after approximately 3 min and the last upright pigs were observed after approximately 3 min, it is reasonable to assume that the pigs had lost consciousness around that time point and well before the concentration reached 80% CO_2_ (after a mean of 7 min). The last movement was observed after 9 min. In Kinsey et al. (2016), all movement had ceased after approximately 10 to 12 min.

Like Arruda et al. (2022), the density of pigs in the container in the current project was based on rules for stocking density for the transport of pigs. This was chosen since the stocking density tables are already integrated and accepted by the Danish veterinary authorities. In addition to a good utilization of the system’s capacity, high stocking density is associated with quicker rises in CO_2_ concentrations (due to the bodies of the pigs filling part of the container volume), but higher stocking densities also increase the risk of dangerous interactions among the animals (Fiedler et al., 2014). Since we put emphasis on avoiding unnecessary stress among the pigs, we chose to use the stocking density for long transports where the pigs have more space than for short transports.

## CONCLUSION

The system described for depopulation of pigs in emergency situations with CO_2_ functioned as intended and no pigs survived at 4 depopulation events. The exact time for loss of consciousness could not be determined due to technical challenges but the system’s capacity met the AVMA’s recommended CO_2_ delivery rate of 10% to 30% of the chamber volume per min. On average, it took 7 min to reach 80% CO_2_ concentration in 4 containers loaded with finishers. By keeping the container tarpaulin closed, the 80% CO_2_ concentration was maintained for at least 10 min after the CO_2_ supply ended. This extended exposure time can be used for transport during emergency depopulation events and contributes to a longer exposure time. Operators should practice on empty containers before depopulation of live pigs.

## Supplementary Material

txaf034_suppl_Supplementary_Materials_1
