# Supplementary material for: Technical note: Construction of a CO2 supply system for depopulation of pigs in a container
Source: Transl Anim Sci. 2025 Mar 11;9:txaf034. doi: 10.1093/tas/txaf034 (PMC12012665; doi:10.1093/tas/txaf034)
Supplement: txaf034_suppl_Supplementary_Materials_1 [file txaf034_suppl_supplementary_materials_1.pdf]

Supplemental Material

Construction of a CO_2 supply system for depopulation of pigs in a container

This supplemental material provides a description of a container (130 cm x 235 cm x 600 cm) with related component list for a concept for depopulation of larger amounts of pigs by use of CO_2.

Heating of CO_2 gas cylinders

For a container measuring 600 × 130 × 235 cm inside, 2 CO_2 batteries of 8 cylinders each were used. Since the cylinders contained liquid CO_2 at a pressure of approx. 50 bar, the gas had to be evaporated from the liquid phase before it could be transferred to the container. This process requires energy. Additionally, gas becomes hot when compressed, but cold if the pressure drops. Therefore, to avoid a large temperature drop during filling of the container (the gas pressure was reduced from 50 bar to 0 bar), the gas must be heated. Fig. 1 illustrates how the energy consumption will cool down the equipment. Fig. 1 (left) also shows 3 thermo-sensors that were attached to each cylinder at 3 levels to measure the temperature at different gas flows.

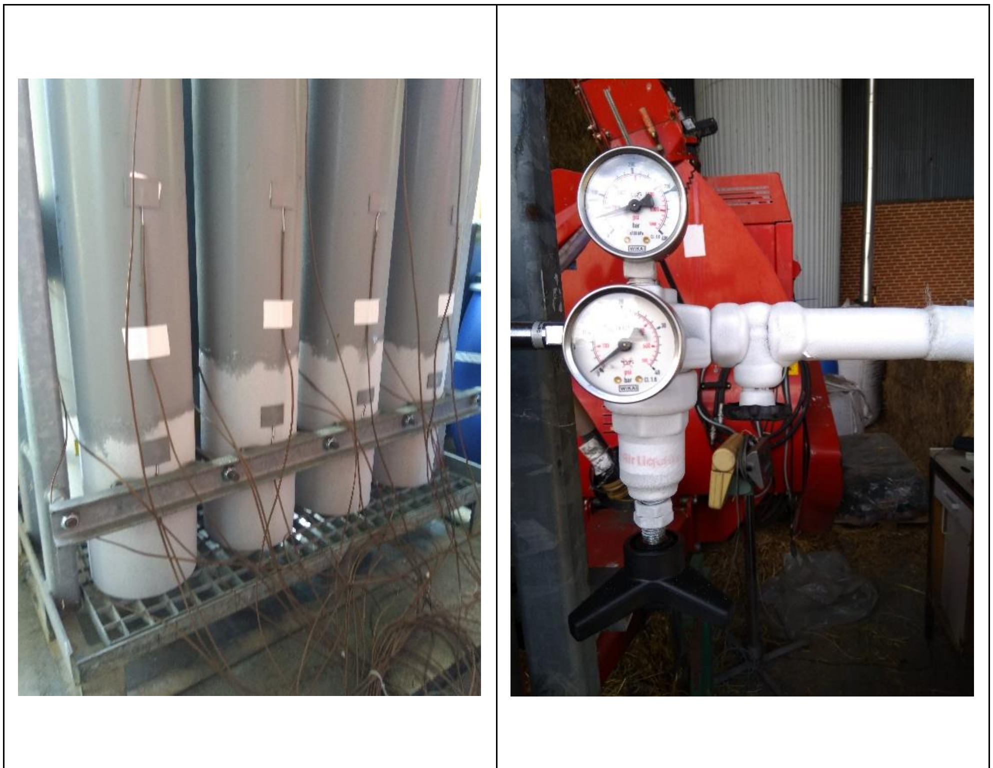

Figure 1. *Frost-coated cylinders (left) and a gas regulator (right) after* CO_2 *supply under normal temperature conditions. Three thermo-sensors are placed at three levels on each cylinder (left).*

The heating requires approx. 24 kW for evaporation of an amount of CO_2, which corresponds to filling up 20% of the container's volume per min. As the heating system for 2 CO_2 batteries has a maximum power of 12 kW and there are some heat losses, the gas supply can only run for about 25% of the time to avoid freezing of the cylinders.

Radiant panels (EUROM Q time 1500 terrace heater; EUROM, Kokosstrat 20, 8281 JC, Genemuiden, Netherlands) was used to heat the CO_2 batteries (Fig. 2). The radiant panels were placed under an open grate that supports the gas cylinders. The radiant panels had 3 performance levels of 500 W, 1000 W and 1500 W, respectively. Here, we used 1500 W per panel, each panel heating two cylinders, corresponding to 6 kW per battery.

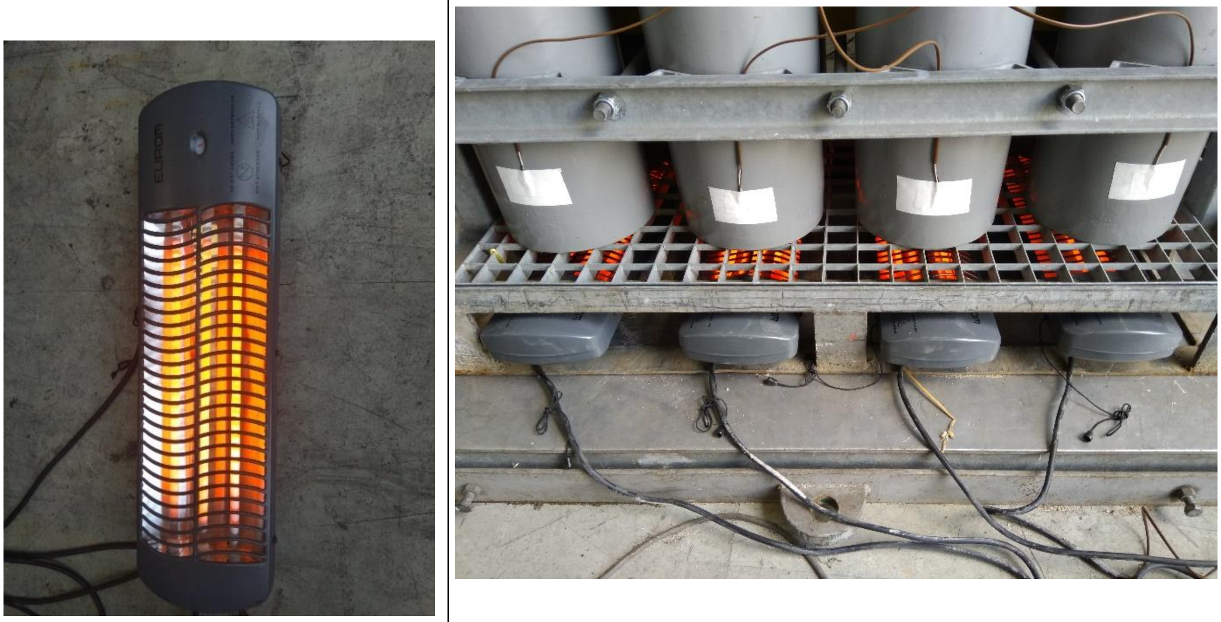

Figure 2. *A radiant panel (left) and a CO_2 cylinder battery heated by 4 radiant panels (right) to heat the gas cylinders enough to evaporate CO_2 from the liquid phase.*

The gas flow was calculated based on the weight loss of the cylinders (1 m^3 of CO_2 weighs approx. 2 kg) by placing the CO_2 batteries on a platform scale.

Heating of CO_2

The pressure drop from approximately 50 bar to 0 bar occurs continuously during exhaust through a pressure regulator (HEPAL 25 NG; Air Liquide, Horsens, DK) and a flow valve (TA STAD ½” string regulation valve; Sanistål A/S, Aalborg, DK). To counteract the associated cooling, the system was equipped with two internal heaters (Klingenberg Electronics, Odense, DK; Fig. 3) heating the CO_2 as it passes. Each heating element is nominally 6 kW at a voltage of 400 V. This means that the power drops to 2000 W when the element is connected to a 230 V

power outlet, which corresponds to the need for one battery. Fig. 4 shows how frost on the equipment disappears when cold gas (left) is heated in a 1½" pipe (right).

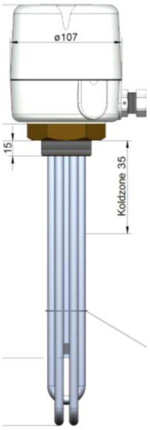

Figure 3. *A 2000 W heater (Klingenberg Electronics, Odense, DK) for heating CO_2 before filling in a container for pig depopulation.*

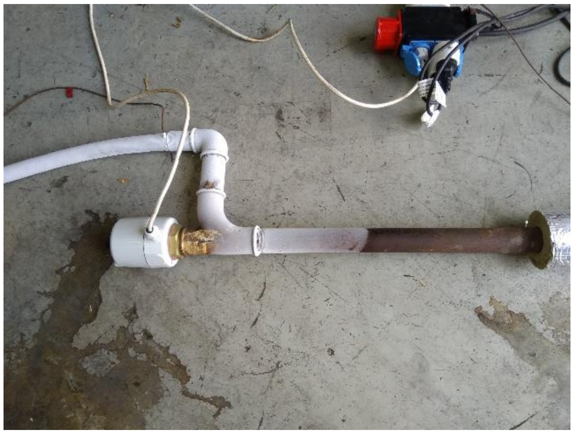

Figure 4. *A 1½" tube that contains a heater and is supplied with CO_2. Frost appears on the left side of the tube before cold CO_2 is heated during blow-through.*

To avoid overheating, the 2000 W heaters (Fig. 5.1) were equipped with a safety thermostat (range 50 – 500°C). The thermostats must be set low, but still high enough to ensure they do not switch off when CO_2 flows. The heaters were retrofitted with indicator lights to show when they are switched on. The heaters ensure that CO_2 is heated by approx. 65 °C, which means that CO_2 is approx. 0 °C when it is blown out into the container. The present method does not create fog in the container. The heated gas not only ensures a fast exhaust speed but also prevents pigs from getting frostbite.

To make the equipment easy to handle, the heating system is built on a EUR pallet (800 mm × 1,200 mm). The piping around the heating elements consists of 1½" galvanized iron pipe. Where CO_2 exits the system (Fig 5.4; white piece) consists of a transition piece between the 1½" thread of the iron pipe and a simple drainpipe for a kitchen sink (50 mm).

Monitoring

To monitor the process, temperatures for CO_2 heating are read at a control panel (Fig 5) also containing two electronic thermostats for each CO_2 battery (Fig. 5.6). Fig. 5.2 shows the gas pipe from the cylinder battery. The temperature of the heated gas is controlled by electronic thermostats (RS Pro 35 x 77 mm ON/OFF Thermostat, NTC, Single Output, RS no. 124-1054; RS Components). The thermostats have a remote sensor (Fig. 5.5) and are connected to a 20 A relay, which cuts off the power to the heater when the gas temperature exceeds 10 °C. The temperature of the heated CO_2 can be set and read on the thermostat's display (Fig. 5.6). Fig. 5.7 marks where the temperature of the heated gas cylinders can be read and set.

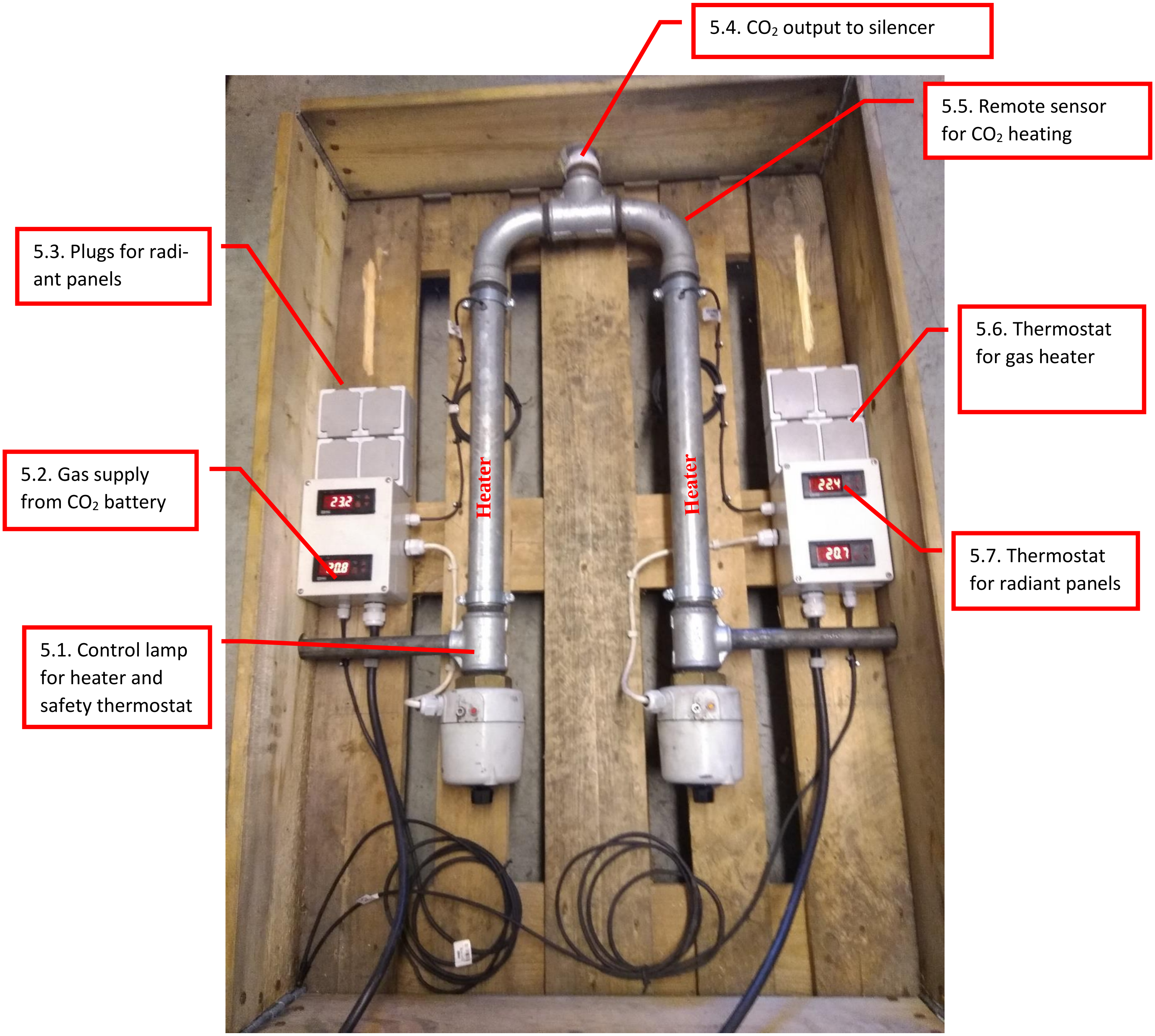

Figure 5. *Heating system for two CO_2 batteries, built on a pallet. 5.1. Control lamp for heating element with associated safety thermostat (illuminates when the heating element is switched on), 5.2. CO_2 supply from cylinder battery, 5.3. Sockets for radiant panels, 5.4. Output for heated CO_2 that is transferred to the system's silencer, 5.5. Remote sensor for observing the temperature of the heated CO_2, 5.6. Thermostat display for reading the temperature (°C) of CO_2 heated with the heater, 5.7. Thermostat display for reading the temperature (°C) of the gas cylinders, heated with the radiant panels placed under the CO_2 batteries.*

Pressure regulator

The CO_2 batteries are equipped with a pressure regulator (HEPAL 25 NG, Air Liquide, Horsens, DK; Fig. 6.1). To ensure a certain back pressure after the regulator, the pressure regulator is built together with a ½” flow control valve (Fig. 6.2). The opening degree of the flow valve can be read on the handle. At the end of the flow valve, an angle is attached with a transition to a 3/4" nipple tube (Fig. 6.3), which functions as a hose connection for a 1" rubber hose for further transport of CO_2 (Fig. 6.4).

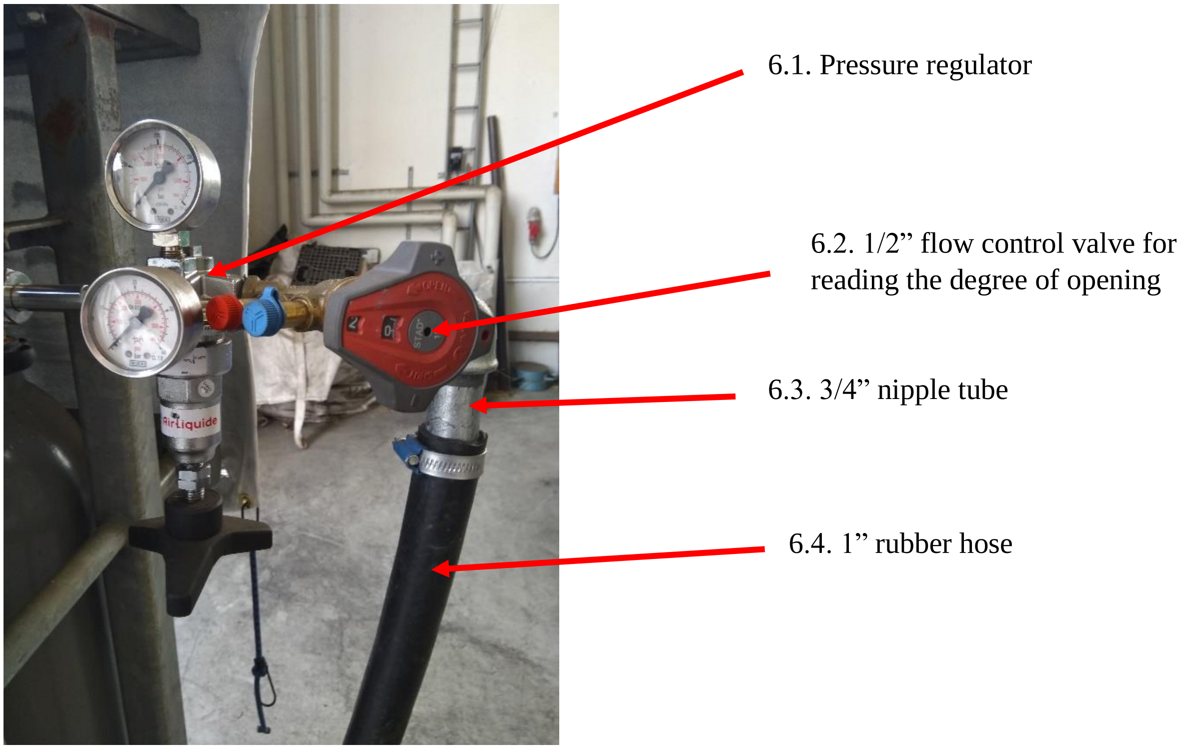

Figure 6. *Pressure regulator and flow control valve attached to a CO_2 cylinder battery.*

Electricity supply

Each CO_2 battery receives its own 16 A power supply through a 5-pin CEE plug. For each battery, there are two double sockets (Schuko) for the radiant panels. The double plug-in connectors

match the plugs on the radiant panels. Each of the double sockets and the thermostat for gas heating is connected to a separate phase to provide an (approximately) even distribution of the current.

Thermostats for the radiant panels

Temperatures above 20 °C will increase the gas pressure of the cylinders when full, entailing a risk of unstable pressure regulators. To avoid overheating of the gas cylinders, they are equipped with an electronic thermostat (RS Pro 35 x 77 mm ON/OFF Thermostat, NTC, Single Output, RS no. 124-1054; RS Components, Fig. 5.6). The thermostats have remote sensors and are connected to a 20 A relay, which cuts off the power to the radiant panels when the cylinder temperature exceeds 15 °C. The remote sensor is taped approx. 1/4 from the bottom of one of the middle cylinders (Fig. 7). The temperature can be set and read on the thermostat's display (Fig. 5.7).

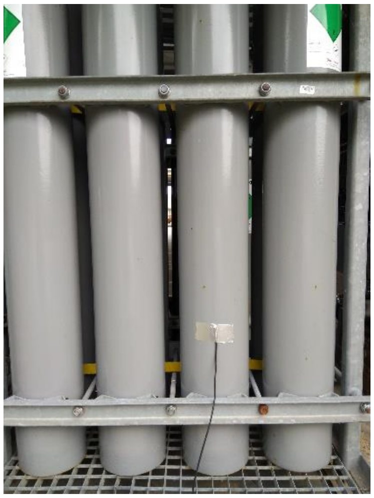

Figure 7. *A remote sensing thermostat attached approx. 1/4 from the bottom of a CO_2 gas cylinder for CO_2 supply to a container for depopulation of pigs. The thermostat prevents overheating of the gas cylinders, which are heated with radiant panels.*

Silencer

The CO_2 leaves the flow valve with a loud hissing sound, which is amplified in the piping system. To limit stress among the pigs, the system is equipped with a silencer (Fig. 8) between the CO_2 heaters and the gas hose leading to the container. The silencer consists of a 1 m long 110 mm drainpipe lined with a Rockwool pipe bowl (48 x 30 mm; Rockwool, Hedehusene, DK). Since the outer diameter of the pipe bowl is 108 mm, it is necessary to cut a few mm of the outer side to make it fit in the plastic tube.

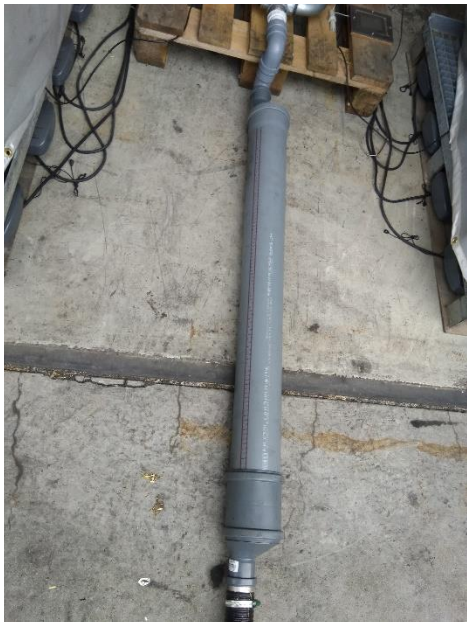

Figure 8. *A silencer for a container system for depopulation of pigs. The silencer consisting of a Rockwool tube bowl attached to a piping system for CO_2 supply.*

Shielding of gas cylinders

Wind and cold weather can easily cool the gas cylinders, which makes it necessary to establish a shielding. The shielding must be a non-combustible material as it is in close contact with the radiant panels. In the present project, a shield made of "welding curtains" was used, which consists of fibre glass (Fig. 9). The curtain measured 2 x 3 m and was provided with holes along the edge, which enable fastening with strips. The curtain must not go all the way to the floor, as there must be free air passage for the radiant panels. Before the welding curtain is drawn around the batteries, it is recommended to cover any sharp corners to protect the fibre glass from breaking.

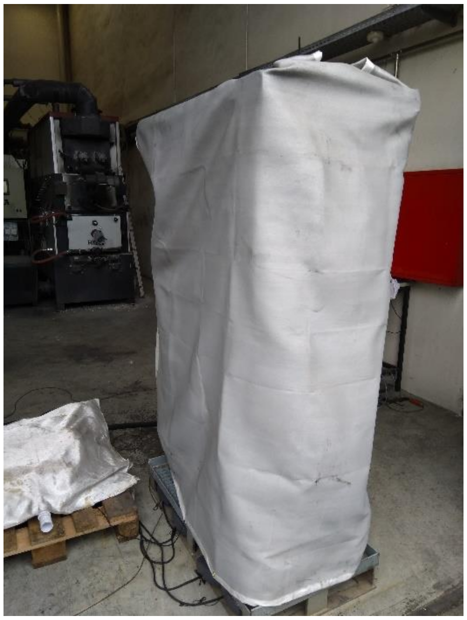

Figure 9. *Non-flammable welding curtain placed around a* CO_2 *cylinder battery to protect against cooling.*

Gas hose for heated gas

From the silencer, the CO_2 is led to the container via a flexible 2” slurry hose, suction/pressure hose (Luisiana SE Ø50 with white PVC spiral; KRAMP, Leek, Groningen Netherlands) which is connected to the inlet pipe in the container using a 2” Bauer quick coupling (Fig. 10.1). Inside the container, the slurry hose goes into a stainless-steel pipe (110 mm), which is mounted in the corner of the container using four strong magnets (Fig. 10.2; Pot Magnet, Ferrite, W/D: 80mm, RS no. 667-9996; RS Components). The steel pipe opens approx. 13 cm above the bottom of the container. As CO_2 is heavier than atmospheric air, it predominantly lies at the bottom of the container, ensuring that CO_2 is supplied from below. As the steel pipe has twice the diameter of the gas hose, it reduces the CO_2 velocity to approx. ¼, which minimizes mixing with atmospheric air. However, there is still some mixing of CO_2 and atmospheric air, especially due to movements of pigs.

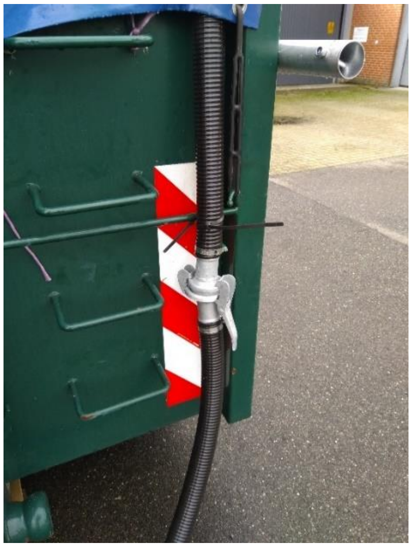

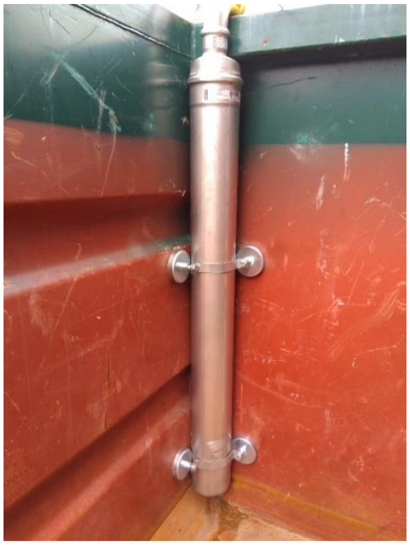

Figure 10. A 2" slurry hose connected to an inlet pipe with a quick connection in a container for depopulation of pigs with CO_2 (left). Inside the container, the slurry hose goes into a stainless-steel pipe, which is mounted with 4 strong magnets in the corner of the container (right).

Sealing of the container

Since the container used was fully welded, there will only be leaks around the tailgate. These were sealed by sticking double rubber gaskets, D-profile 21 x 15 mm and D-profile 12 x 10 mm, (Profilageret ApS, Kolding, Denmark) on the vertical edges (Fig. 11). On the lower vertical edge of the door, it was only necessary to use a single seal (Fig. 12). Sealing of the container's bottom plate was omitted, as it is easily damaged when pigs are loaded and unloaded.

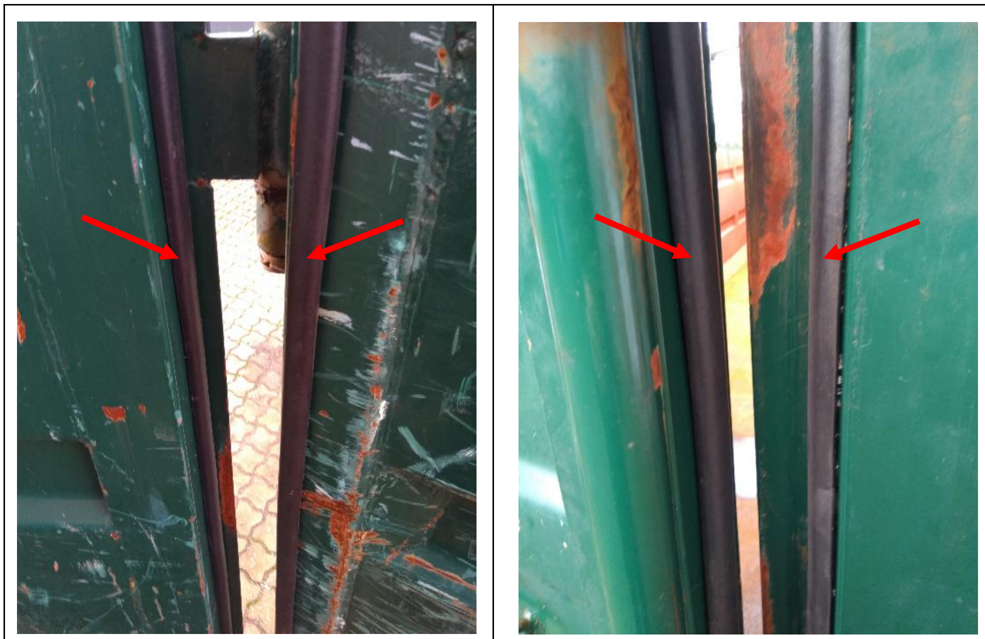

Figure 11: *A rubber seal for sealing a container against CO_2 leaks. Double rubber seals are glued to the rear door of the container's hinge sides (left) and to the middle of the door (right).*

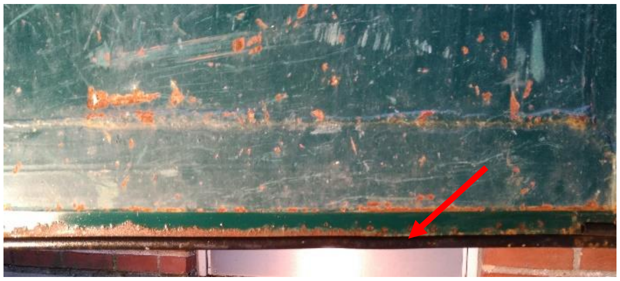

Figure 12. *A rubber seal for sealing a container against* CO_2 *leaks. A single rubber gasket is glued to the lower edge of the door. The bottom of the container has no rubber seal.*

The pipe with CO_2 is led in over the edge of the container, but under the tarpaulin, which acts as a cover. To seal around the gas pipe, it is passed through a triangular piece of foam rubber (Fig. 13). The foam rubber is a simple "mason's sponge".

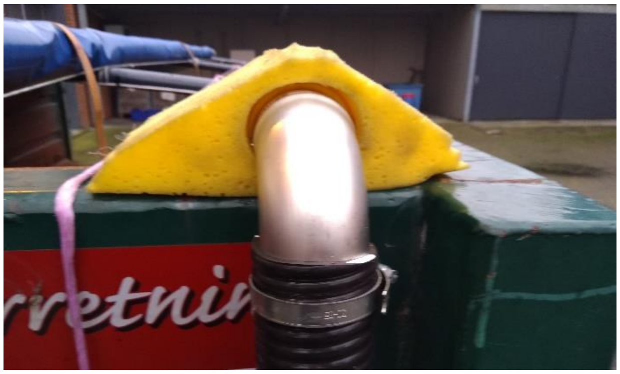

Figure 13. *Pipe for blowing in* CO_2*. The pipe is placed between the upper edge of the container and a tarpaulin, which is used as cover. A foam rubber sponge functions as a seal between the pipe, the tarpaulin and the container.*

Comments

During CO_2 supply, the pressure in the CO_2 batteries must be monitored, as the batteries have the highest pressure when full, and the drop in pressure will cause the temperature of CO_2 to drop. The pressure must not fall below approx. 10 bars since a (too) low pressure results in a (too) low CO_2 flow. The pressure in the batteries can be maintained by increasing the setpoint on the thermostat (Fig. 5.7) for heating the CO_2 batteries with 1.5 °C for each filling of the container. The less CO_2 left in the batteries, the more the temperature drops during the supply. Therefore, the CO_2 batteries are difficult to empty completely. To some degree, this challenge can be solved by first using a "normal" flow for approx. 3 min. until the pigs are unconscious and no sounds are heard from the container. After that, the flow can be lowered, and the operator can continue the process with a low CO_2 flow for the next 12-15 min. This will help the radiant panels keep the CO_2 batteries sufficiently warm.

It is recommended as a minimum to continuously measure the CO_2 concentration at least in one place in the container at a height of approx. 75 cm, so the supply can be terminated at 80% CO_2. If the CO_2 meter fails, CO_2 should be supplied at normal flow for a minimum of 8 min.

Component list

Number	Name	Type	Supplier
2	Pressure regulator	HEPAL25 NG	Air Liquide[1]
2	Control valve	TA STAD ½” strengreguleringsventil	Sanistaal[2]
2	Rubber hoses	1” x 3 m rubber hoses	Own stock
8	Radiant panels	Eurom terrassevarmer, HN nr. 19120	Harald Nyborg[3]
2	Gas heater	Elpatron CO2	Klingenberg Electronics[4]
4	Thermostats	Thermostat, NTC	RS Components[5]
4	Sensors for thermo-stats	Air probe, RS nr. 124-1081	RS Components[5]
2	Relays for the do.	DPNO Effektrelæ, RS nr. 511-1111	RS Components[5]
2	Cabinets for the do.	ABS Universalkabinet, RS nr. 201-0191	RS Components[5]
4	Magnets	60 kg kop-magneter, RS nr. 667-9996	RS Components[5]
1	Connection pipe 1½”	50 mm connection to sink	Jem & Fix[6]
4	Double plugs	Double Schuko plugs	Elvvs.dk[7]
2	16 A plug	16 A CEE plug	Elvvs.dk[7]
1	110 mm pipe	1 m 110 mm stainless pipe	Elvvs.dk[7]
1	Reduction	110-50 mm stainless pipe reduction	Elvvs.dk[7]
2	Bends	50 mm stainless 88° bends	Elvvs.dk[7]
1	50 mm pipes	500 x 50 mm stainless pipe	Elvvs.dk[7]
2	1½” bends	1½” galvanized bend. i/o	Elvvs.dk[7]
1	1½" Tee	1½" galv. Tee	Elvvs.dk[7]
2	1½” Tee	1½-3/4-1½” Tee	Elvvs.dk[7]
4	¾” nipple pipe	¾” x 60 mm galvanized nipple pipe	Elvvs.dk[7]
1	1½" nipple pipe	1½" x 50 mm galvanized nipple pipe	Elvvs.dk[7]

2	Pipe pieces	1½” x 500 mm galvanized pipe	The local blacksmith
2	Angles	3/4 -½” galvanized angle	Elvvs.dk[7]
4	Pipe carriers	1½" galvanized pipe carriers	Elvvs.dk[7]
1	Pipe bowl	48 x 30 mm Rockwool pipe bowl	Elvvs.dk[7]
1	110 mm pipe	HTP Ø110 mm x 1 m gray drainage pipe	Elvvs.dk[7]
2	Reduction	HTP reduction 110-50 mm gray	Elvvs.dk[7]
2	Bend	HTP bend 88°, 50 mm gray	Elvvs.dk[7]
1	Socket	HTP double socket, grey, 110 mm	Elvvs.dk[7]
10 m	Gas hose	Suction/pressure hose Louisiana SE Ø50	Kramp[8]
1	Coupling	2” Bauer coupling male/female	Kramp[8]
2	Welding blanket	2 x 3 m welding blanket	Brand-tech.dk[9]
10 m	Rubber seal	D-profile 21 x 15 mm	Profillageret.dk[10]
6 m	Rubber seal	D-profile 12 x 10 mm	Profillageret.dk[10]

1) Air Liquide Denmark, Uraniavej 6, 8700 Horsens, DK; 2) Sanistaal, Søren Frichs Vej 44A, 8230 Åbyhøj, DK; 3) Harald Nyborg, Christiansminde 1, 8800 Viborg, DK; 4) Klingenberg Electronics, Kronprinsensgade 8, 5000 Odense,DK; 5) RS Components, Vennershåbvej 4, 8800 Viborg, DK; 6) Jem & Fix, Skomagervej 12, 7100 Vejle, DK; 7) Elvvs.dk, Søndergårdsvej 44, 4640 Faxe, DK; 8) Kramp Denmark, Kobbervej 6 6900 Skjern, DK; 9) Brand-tech.dk, Håndværkerbyen 13, 2670 Greve, DK; 10)Profillageret.dk, Platinvej 61, 6000 Kolding, DK
